# Individual crypt genetic heterogeneity and the origin of metaplastic glandular epithelium in human Barrett’s oesophagus

**DOI:** 10.1136/gut.2007.143339

**Published:** 2008-02-27

**Authors:** S J Leedham, S L Preston, S A C McDonald, G Elia, P Bhandari, D Poller, R Harrison, M R Novelli, J A Jankowski, N A Wright

**Affiliations:** 1 Histopathology Unit, Cancer Research UK, London, UK; 2 Institute of Cell and Molecular Sciences, St Bartholomew’s and Royal London School of Medicine and Dentistry, Queen Mary University, London, UK; 3 Department of Clinical Pharmacology, University of Oxford, Oxford, UK; 4 Department of Gastroenterology Queen Alexandra Hospital, Portsmouth, UK; 5 Pathology Department, Queen Alexandra Hospital, Portsmouth, UK; 6 Pathology Department, Leicester General Hospital, Leicester, UK; 7 Histopathology Department, University College Hospital, London, UK; 8 Digestive Disease Centre, Leicester Royal Infirmary, Leicester, UK

## Abstract

**Objectives:**

Current models of clonal expansion in human Barrett’s oesophagus are based upon heterogenous, flow-purified biopsy analysis taken at multiple segment levels. Detection of identical mutation fingerprints from these biopsy samples led to the proposal that a mutated clone with a selective advantage can clonally expand to fill an entire Barrett’s segment at the expense of competing clones (selective sweep to fixation model). We aimed to assess clonality at a much higher resolution by microdissecting and genetically analysing individual crypts. The histogenesis of Barrett’s metaplasia and neo-squamous islands has never been demonstrated. We investigated the oesophageal gland squamous ducts as the source of both epithelial sub-types.

**Methods:**

Individual crypts across Barrett’s biopsy and oesophagectomy blocks were dissected. Determination of tumour suppressor gene loss of heterozygosity patterns, *p16* and *p53* point mutations were carried out on a crypt-by-crypt basis. Cases of contiguous neo-squamous islands and columnar metaplasia with oesophageal squamous ducts were identified. Tissues were isolated by laser capture microdissection and genetically analysed.

**Results:**

Individual crypt dissection revealed mutation patterns that were masked in whole biopsy analysis. Dissection across oesophagectomy specimens demonstrated marked clonal heterogeneity, with multiple independent clones present. We identified a *p16* point mutation arising in the squamous epithelium of the oesophageal gland duct, which was also present in a contiguous metaplastic crypt, whereas neo-squamous islands arising from squamous ducts were wild-type with respect to surrounding Barrett’s dysplasia.

**Conclusions:**

By studying clonality at the crypt level we demonstrate that Barrett’s heterogeneity arises from multiple independent clones, in contrast to the selective sweep to fixation model of clonal expansion previously described. We suggest that the squamous gland ducts situated throughout the oesophagus are the source of a progenitor cell that may be susceptible to gene mutation resulting in conversion to Barrett’s metaplastic epithelium. Additionally, these data suggest that wild-type ducts may be the source of neo-squamous islands.

Barrett’s oesophagus is the replacement of the normal oesophageal stratified squamous epithelium with metaplastic glandular epithelium in response to inflammation and ulceration provoked by duodeno-gastroesophageal reflux.^1^ Oesophageal adenocarcinoma can arise from progression through a metaplasia–dysplasia–carcinoma sequence (MCS), and the presence of Barrett’s oesophagus increases the risk of oesophageal adenocarcinoma by 30- to 40-fold.^2^

Serial biopsies and molecular analysis of a cohort of patients with Barrett’s oesophagus has enabled researchers to study the evolution of common tumour suppressor gene mutation patterns as the MCS progresses. These longitudinal clonal ordering studies have shown that genetic and epigenetic inactivation of *cyclin-dependent kinase N2* (*p16*) and genetic inactivation of *TP53* (*p53*) tumour suppressor genes occur early in the MCS^3^ with 88% of pre-dysplastic Barrett’s oesophagus tissue having a detectable *p16* lesion.^4^ The demonstration of clonal *p16* and *p53* lesions throughout long lengths of Barrett’s oesophagus^4^ ^5^ suggests a common precursor lesion that undergoes clonal expansion, and has led to the proposal that the MCS progresses as a consequence of sequential tumour suppressor gene inactivation causing selective growth advantages. Growth advantages result in preferential expansion of a mutated clone and a mutation is said to have “gone to fixation” when it expands throughout an entire field, extinguishing all competing clones. A “selective sweep” is the process of natural selection driving a mutation to fixation.^6^ ^7^ It has been suggested that loss of each of the two *p16* alleles predisposes to a selective sweep, and that *p16* mutation fixation occurs early in the progression of Barrett’s oesophagus.^6^ The demonstration of similar *p16* loss of heterozygosity (LOH), methylation and point mutation patterns in biopsy material taken from different levels of long Barrett’s oesophagus segments supports widespread clonal expansion and fixed mutations.^4^ However, phenotypic and genotypic heterogeneity has also been described in some Barrett’s segments^4^ ^5^ and clonal diversity in Barrett’s segments has recently been shown to be associated with progression to adenocarcinoma.^8^

Despite years of active research the histogenesis of Barrett’s oesophagus has never been demonstrated. Different theories have been proposed: proximal migration of the gastric cardia; re-differentiation of the squamous epithelium and colonisation of cells from the oesophageal gland ducts.^9^ ^10^ Similar questions remain regarding the origin of neo-squamous islands that can arise within fields of Barrett’s tissue after acid suppression or endoscopic ablative therapy.^11^ ^12^ Paulson *et al*^13^ demonstrated that these squamous islands were usually genetically wild-type despite being surrounded by mutated Barrett’s tissue. They excluded encroachment of adjacent normal squamous epithelium by only including patients who developed isolated squamous islands, but were unable to determine the source of the genetically normal tissue that may have an important clinical role in re-epithelisation after treatment for Barrett’s oesophagus.

To date, most clonality studies have been carried out on heterogenous flow-purified whole biopsy samples. In this work our aims were to (1) study clonality at a crypt-by-crypt level, avoiding problems associated with contaminating normal stroma; and (2) examine the oesophageal gland squamous ducts as the potential source of Barrett’s columnar epithelium and neo-squamous islands.

## MATERIALS AND METHODS

### Tissue and slides

Paraffin-embedded biopsy (six biopsies from five patients) and oesophagectomy blocks (four blocks from patient 1, four blocks from patient 2 and two blocks from patient 3) were obtained from the pathology archives of Leicester General Hospital. Tissue was independently assessed for Barrett’s metaplasia and dysplasia according to British Society of Gastroenterology 2005 guidelines (www.bsg.org.uk), by at least two pathologists. Serial 5 μm sections were cut. Sections 1–3 and 5–7 were mounted onto P.A.L.M. membrane slides (P.A.L.M. Microlaser Technologies, Benried, Germany) and were stained with methylene green. Section 4 was stained with haematoxylin & eosin (H&E).

### Laser capture microdissection (fig 1)

Suitable crypts for dissection were identified using the H&E slide. The same crypts were identified on the slides stained with methylene green. Individual crypt sections from the six serial slides were cut out from the laser capture slides and catapulted into the adhesive caps of eppendorfs using the P.A.L.M. Laser Microdissection system. Where constitutional DNA was required for microsatellite analysis, serial areas of lamina propria were microdissected. Catapulted sections on the cap were immersed in 12 μl of proteinase K solution (Arcturus Bioscience, Mt View, California, USA). After individual crypt dissection residual epithelial tissue was catapulted into a single tube and immersed in 30 μl proteinase K for *p53* gene screening. Negative control tubes containing 12 μl proteinase K solution and no laser capture material were included. Tubes were then centrifuged at 4.5 *g* for 1 min and incubated at 65°C overnight. A 10 min incubation at 95°C denatured the proteinase K and the lysate was then stored at −20°C.

### Immunocytochemistry

Cytokeratin 7 and 13 staining was used to demonstrate glandular and squamous epithelial differentiation respectively.^14^ ^15^ Serial sections of oesophagectomy blocks were cut at 4 μm and mounted on glass slides. Sections were de-waxed and rehydrated by standard methods. Endogenous peroxidase was blocked with 3% H_2_O_2_ in methanol for 10 min. Antigen retrieval was achieved by 10 min microwaving in sodium citrate buffer at pH 6. Slides were incubated in 3% bovine serum albumin in phosphate-buffered saline (PBS) for 15 min. Slides underwent primary antibody incubation with mouse monoclonal antibodies against cytokeratin 7 (1:100 dilution of clone OV-TL; Abcam, Cambridge, UK) or cytokeratin 13 (1:200 dilution of clone AE8; Abcam). This was followed by biotinylated rabbit anti-mouse secondary antibodies before application of a 1:500 dilution of the tertiary layer of peroxidase-conjugated streptavidin (strep-HRP; Dako, Glostrup, Denmark). Each layer was applied for 45 min and three 5 min PBS washes were performed between layers. Sections were then developed with 3,3-diaminobenzidine tetrahydrochloride solution (DAB; Sigma, Poole, UK) for 2 min, followed by rinsing in tap water and light haematoxylin counterstaining. The positive control tissues used were duodenum (CK 7) and tonsil (CK 13). Negative controls underwent all steps but were incubated with PBS instead of the primary antibody solution.

### Nested polymerase chain reaction and sequencing

First and second round primers were designed to amplify exons 5–9 of *p53* and exon 2 of *p16*, using the primer 3 website (MIT, Cambridge, Massachusetts, USA). First round oligonucleotide primer pairs were specifically designed to amplify a region that included the amplicon covered by the primers used in the second round of the polymerase chain reaction (PCR). Primer optimisation determined the optimum reagent concentration and annealing temperature for each primer pair. Primer sequences are tabulated in the supplementary information (supplementary table 1A–C). Both PCR steps were carried out in an Omni PCR UV hood to minimise contamination (Bioquell, Andover, Berkshire, UK). Only products with an uncontaminated negative control tube went forward for sequencing. PCR product was sequenced using BigDye terminator cycle sequencing on an ABI 3100 DNA sequencer (Applied Biosystems, Foster City, California, USA). The sequences obtained were directly compared to the revised Cambridge reference sequence, and any identified mutations were checked against the Catalogue of Somatic Mutations in Cancer (COSMIC) database (www.sanger.ac.uk) (Cambridge, UK). Sequencing was repeated twice from dissection lysate for the oesophageal gland squamous duct work.

### Microsatellite analysis

Three microsatellite markers; D5S346 (*APC* gene), D9S932 (*p16* gene) and D17S786 (*p53* gene), were used for LOH analysis. Constitutionally homozygous markers were scored as non-informative. Forward oligonucleotide primers were labelled at the 5′ end with the carboxy fluorescein (FAM) fluorescent marker (Sigma). PCR amplifications were performed using the LA TAKARA kit (Takara Bio, Shiga, Japan). The PCR product was analysed on an ABI 3100 sequencer (Applied Biosystems) and genotyper 2.5 software (Perkin-Elmer, Boston, Massachusetts, USA). At each marker, loss of heterozygosity was considered present if the area under one allelic peak in the affected crypt was less than 0.5 times or greater than 2 times that of the other allele, after correcting for the relative areas using constitutional DNA (microdissected areas of lamina propria tissue).

### Statistical analysis

The associations between individual crypt point mutations and tumour suppressor gene allelic loss were analysed using the two-tailed Fisher’s exact test run on Prism 4.0 software (Graphpad Software, San Diego, California, USA).

## RESULTS

### Biopsies

Thirty-seven individual crypts and seven squamous islands were dissected from six biopsies taken from five patients. Analysis of individual crypts from biopsy samples allowed detection of LOH patterns in *p16*, *p53* and *adenomatous polyposis coli* (*APC*) tumour suppressor genes which is not demonstrable when the whole biopsy section was digested and analysed. This is likely to be a consequence of a diluting effect of the normal stroma in the whole biopsy sample (fig 2 and supplementary fig 1)

**Figure 1:**
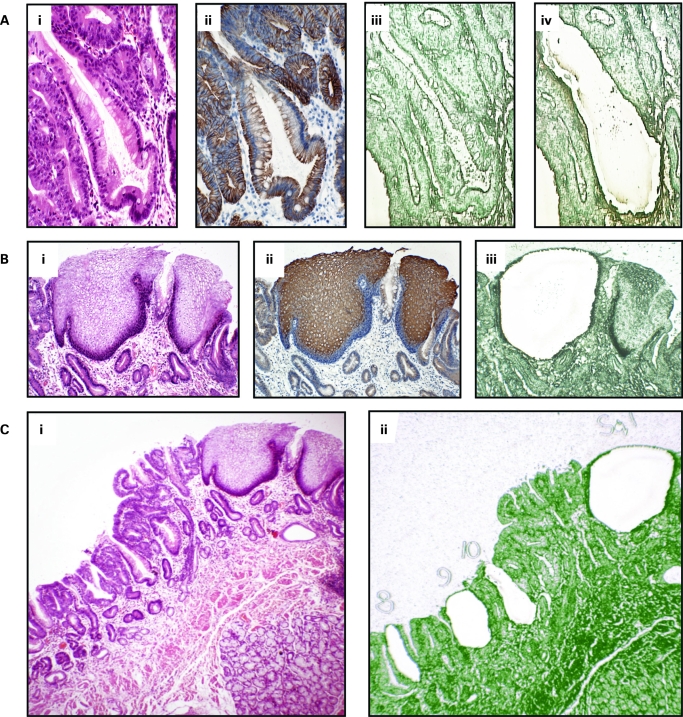
Laser capture microdissection. A(i) Individual crypts were identified using haematoxylin & eosin (H&E) slides A(ii) Cytokeratin 7 immunostaining of serial sections showing glandular differentiation of columnar lined oesophagus. A(iii), (iv) Individual crypts were microdissected from serial sections stained with methylene green and mounted on laser capture slides. B(i) Squamous islands were identified histologically using H&E slides. B(ii) Cytokeratin 13 immunostaining of serial sections showing mature squamous cell differentiation. B(iii) Squamous island after laser capture showing selective dissection of only squamous tissue. C(i), (ii) Individual structures were dissected evenly from across the block and numbered.

**Figure 2:**
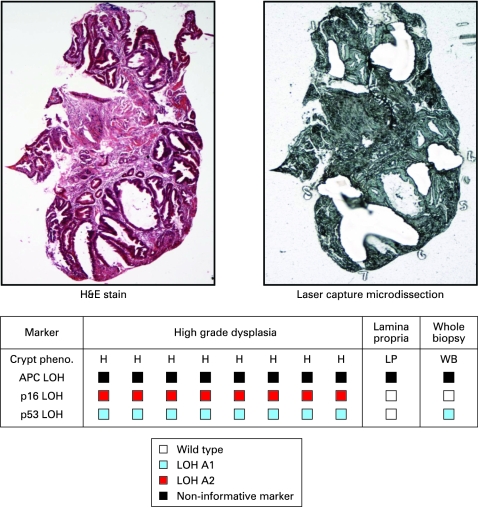
Oesophageal biopsy clonal map. Individual crypts, squamous islands and areas of lamina propria were microdissected from oesophageal biopsies and analysed for tumour suppressor gene loss of heterozygosity (LOH). An unstained serial section of the whole biopsy was then scraped into proteinase K using a clean scalpel blade, and the lysate analysed. LOH of individual alleles is denoted by a blue- or red-coloured box. The table is the clonal map obtained from a single biopsy and each column within the table represents genetic analysis of a single crypt or area of lamina propria. In each case the shortest allele is referred to as A1 and the longest A2. Non-informative markers are denoted by a black box. Individual crypts often demonstrated LOH not detectable on the whole biopsy section lysate probably as a consequence of a diluting effect of wild-type stroma in the whole biopsy lysate. Other biopsy clonal maps are presented in the supplementary information (supplementary fig 1). H&E, haematoxylin & eosin.

### Oesophagectomy blocks

**Figure 3:**
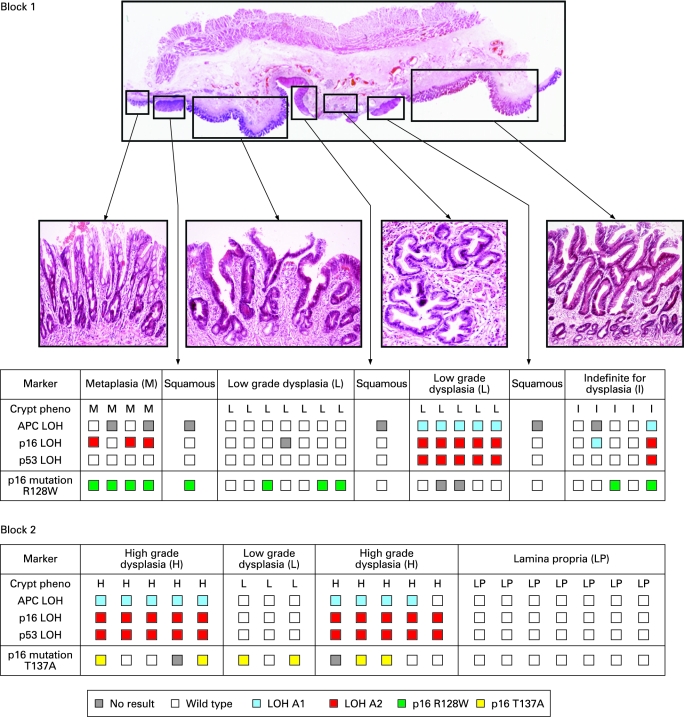
Clonal maps of two blocks from patient 1. Each table is the clonal map for the oesophagectomy specimen pictured and each column within the table represents the genetic analysis of a single crypt, squamous island or area of lamina propria. *p16* point mutations are denoted by green- or yellow-coloured boxes as per the key. White boxes are wild-type. Clonal analysis revealed regional similarities in loss of heterozygosity (LOH) patterns correlating with the observed phenotypic differences; however, there was no evidence of tissue-wide selective sweeps and no fixed founder mutations indicating a common ancestral precursor. Two different, independent *p16* point mutations were identified in the different blocks suggesting at least two distinct clones. Only one squamous island contained a mutation and this is comparable with the results described by Paulson *et al*^13^ Clonal maps from other patients are presented in the supplementary information (supplementary fig 2).

One hundred and twenty-seven individual Barrett’s crypts and 14 squamous islands were dissected from 10 oesophagectomy blocks from three patients. Squamous islands were defined as an area of squamous mucosa seen completely surrounded and engulfed by metaplastic tissue. Suprabasal squamous island immunostaining with cytokeratin 13 antibody demonstrated squamous differentiation. Clonal heterogeneity of LOH patterns and identified point mutations were found in all of the oesophagectomy blocks. The identification of distinct point mutations is a powerful tool for the demonstration of clonality, and one patient had two different *p16* point mutations in crypts from different blocks indicating the presence of at least two separate clones (fig 3). No single fixed mutation was identified throughout every dissected crypt from an entire block, although some *p53* point mutations were present in multiple blocks from the same patient (supplementary fig 2). Individual Fisher’s exact tests were calculated for each point mutation and allelic loss. The calculated p-values are tabulated and show a significant association between *p53* point mutations and loss of any of the three alleles. No association was seen between *p16* point mutation and tumour suppressor gene LOH.

**Table 1:** Fisher’s exact tests showing association between each point mutation and individual allelic loss

Mutation	*p53* LOH	*p16* LOH	*APC* LOH
*p53* point mutation	p = 0.0034	p = 0.0003	p<0.0001
*p16* point mutation	p = 0.42	p = 0.21	p = 0.08

LOH, loss of heterozygosity.

### Oesophageal gland squamous ducts

**Figure 4:**
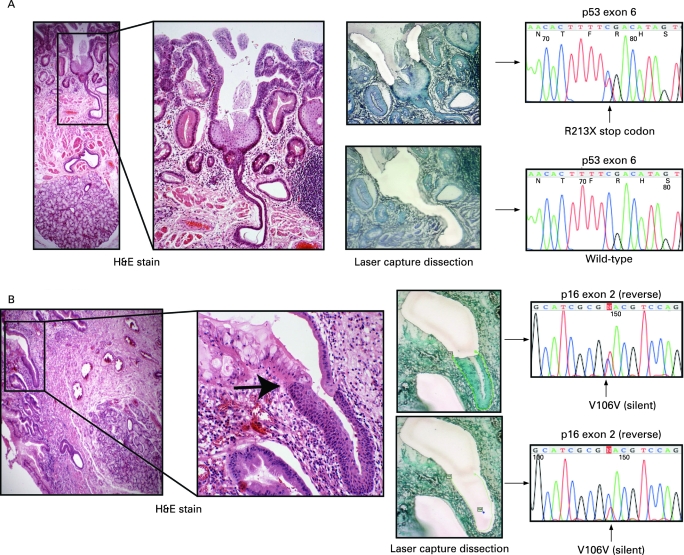
(A) Oesophageal gland squamous ducts give rise to neo-squamous islands. The haematoxylin & eosin (H&E) slides show a squamous island originating from an oesophageal gland squamous duct and encroaching onto a field of Barrett’s dysplasia. Dissection of the continuous dysplastic tissue revealed a *p53* non-sense mutation, which was not present in the separately dissected squamous tissue and squamous duct. It has been previously noted that squamous islands are often associated with oesophageal glands,^16^ and this demonstrates the oesophageal gland duct as the source of these wild-type islands. (B) Oesophageal gland squamous ducts give rise to metaplastic columnar epithelium. The H&E slides show a metaplastic glandular crypt arising from a contiguous squamous duct with a clear transition from squamous to columnar epithelium (black arrow). The same mutation was found in both the squamous and columnar epithelial tissue suggesting a clonal origin.

All but one of the dissected squamous islands were wild-type, despite often being surrounded by fields of mutated Barrett’s crypts. Wild-type squamous islands were seen overgrowing mutated deep Barrett’s oesophagus crypts as a thin surface layer and this is consistent with previous results, particularly after attempts at ablative treatment of Barrett’s.^11^ Moreover, it was common to find squamous islands situated overlying deeper oesophageal glands. In one case a squamous island was seen arising from an oesophageal gland duct encroaching onto a field of Barrett’s oesophagus (fig 4A). Microdissection of the surrounding Barrett’s epithelium revealed a *p53* non-sense point mutation; however, the underlying squamous island and contiguous oesophageal gland squamous duct were *p53* wild-type, indicating the presence of a different clone to the surrounding Barrett’s dysplasia. In three tissue blocks it was also possible to identify oesophageal squamous duct epithelium in continuity with metaplastic Barrett’s crypts similar to the findings of Coad *et al.*^16^ In one case careful microdissection of the metaplastic epithelium revealed a silent point mutation in exon 2 of *p16*, which was also seen in the separately dissected squamous duct (fig 4B). The mutation is non-coding and is therefore unlikely to be the founder mutation responsible for metaplastic transition but does serve as a useful clonal marker showing a clonal origin of both epithelial types and suggesting that metaplastic epithelium arises from squamous duct origin. The same mutation was also detected in the oesophageal gland acini suggesting bi-directional flow of mutated cell progeny similar to that seen in the Brunner’s gland.^17^

## DISCUSSION

This study has improved upon previous clonality studies in Barrett’s oesophagus by studying the clonal origin of individual crypts rather than purified whole biopsy specimens. We have shown here that single biopsies can be phenotypically and genotypically heterogeneous, and analysis of whole biopsy samples masked some of the mutations picked up on individual crypt analysis. Thus, genetic analysis of whole biopsy samples, even those that are well targeted, may not detect all mutations or clones, throwing into doubt their use as surveillance biomarkers in Barrett’s oesophagus since minority clones in the sample may not be detected.

Dissection across large oesophagectomy blocks also revealed considerable phenotypic and genotypic heterogeneity in all cases. *p16* point mutations were limited to single blocks and were not significantly associated with loss of any alleles; however, there was a significant association (Fisher’s exact test p<0.004) between *p53* point mutations and allelic loss of all three tumour suppressor genes. This is consistent with functional loss of the cell cycle checkpoint activity of TP53 protein, which is then permissive for widespread, large-scale genetic changes. Additionally, *p53* point mutations, although not seen in every crypt, were often present in multiple blocks from a single oesophagectomy specimen, suggesting widespread and far-reaching clonal expansion as a consequence of the strong selective advantage that *p53* loss would provide (supplementary fig 2). This is similar to the findings by Prevo *et al*,^18^ but our individual crypt analysis technique also allows detection of competing p53 wild-type clones, which may have been masked in whole biopsy samples.

Phenotypic and genotypic heterogeneity in Barrett’s tissue has been well described,^4^ ^5^ ^8^ including patients with multiple distinct *p53* point mutations.^18^ This heterogeneity has previously been explained by clone bifurcation with genetic divergence of an original single population; however, such bifurcation importantly assumes the presence of an original founder, fixed mutation which should still be able to identify all crypts, despite subsequent genetic divergence. Using our crypt-by-crypt analysis we have failed to show a single founder mutation present in every crypt throughout an entire block, including *p16* inactivation. Bi-allelic loss of *p16* in some crypts means that epigenetic methylation is unlikely to be an undetected founder mutation. *p16* inactivation by allelic loss, point mutation or methylation is one of the proposed earliest lesions in the Barrett’s MCS pathway and is said to predispose towards a selective sweep.^6^ We could find no evidence of fully fixed mutations: on the other hand regional LOH and point mutation patterns could be identified. This suggests sweeps of clonal expansion may be localised amongst multiple independent clones, rather than a single founder mutation sweeping through an entire Barrett’s segment to fixation.

It has been proposed, but never proven, that submucosal oesophageal gland ducts may be the origin of metaplastic tissue in Barrett’s oesophagus.^10^ ^16^ ^19^ These results support this hypothesis by demonstrating a *p16* point mutation originating in microdissected squamous duct tissue that was also found in the adjoining metaplastic crypt. The presence of an identical mutation in the two different epithelial types is strong evidence to suggest that the origin of the metaplastic tissue in human Barrett’s oesophagus is a progenitor located in the oesophageal gland squamous ducts. Additionally, the presence of a wild-type squamous island seen emerging from a wild-type squamous duct in the midst of, and completely surrounded by, a *p53* mutant field, strongly indicates a new clone development. This supports the hypothesis that neo-squamous islands can arise de novo from glandular tissue after Barrett’s ablation therapy,^20^ and extends the findings by Paulson *et al*^13^ by showing that these wild-type islands arise from non-mutated squamous duct tissue.

**Figure 5:**
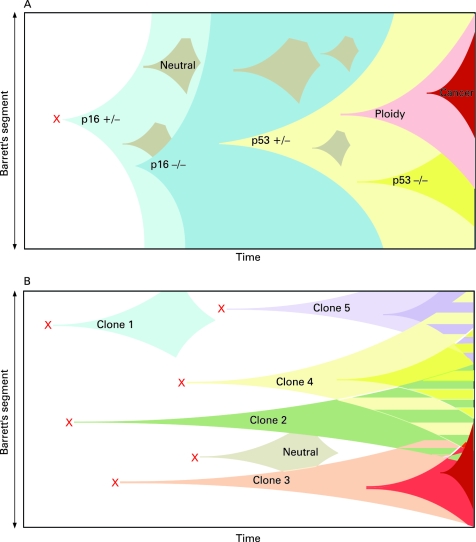
Clonal evolution models in Barrett’s oesophagus. (A) The current model of clonal evolution adapted from Maley *et al*.^6^ A founder mutation (red cross) occurs in a single progenitor and provides a growth advantage that predisposes to a selective sweep. Successive selective sweeps result in progression along the metaplasia–dysplasia pathway. Clone bifurcation is responsible for clonal heterogeneity in this model. (B) Newly proposed model of evolution based on the mutation of multiple progenitor cells situated in the oesophageal gland squamous ducts located throughout the length of the oesophagus (red crosses). Multiple independent clones then arise which evolve separately. The presence of multiple different clones gives rise to a mosaic interdigitating clonal pattern of the Barrett’s segment represented here by the striped areas.

The demonstration of multiple competing independent clones and the identification of the origin of Barrett’s oesophagus from the oesophageal gland squamous duct, structures present throughout the length of the oesophagus, allows the development of a new model of clonal evolution in Barrett’s oesophagus. We suggest that duodeno-gastro-oesophageal reflux-induced ulceration and inflammation can induce tumour suppressor gene mutations in some of the stem cell populations located in oesophageal gland squamous ducts. This gives rise to multiple distinct clones of metaplastic tissue that then compete to colonise the oesophagus, creating a mosaic pattern of clones across the segment. Clonal expansion of populations with greater selective advantage leads to dominant and widespread clones; however, no single mutation is fixed since competing wild-type clones are also identifiable. Non-mutated squamous ducts are likely to be the source of wild-type squamous islands (fig 5). These data are unable to rule out extension of mutated tissue from the oesophageal–gastric junction; however, we propose that the oesophageal gland ducts should be considered as an alternative or additional source of the multiple independent clones we have demonstrated here.

The cell biology of the oesophageal epithelium has been inadequately studied despite the high mortality and increasing incidence of cancer at this site. The interpapillary basal layer has been suggested as a possible stem cell zone^21^ but the demonstration of Barrett’s metaplasia arising from oesophageal squamous ducts raises an interesting analogy with the skin, another stratified squamous epithelium. In the epidermis, stem cells not only reside in the interfollicular epithelium but also in associated deeper structures; specifically, the bulge region of the hair follicle.^21^ The clinical significance of a possible oesophageal gland duct stem cell niche, both as the source of Barrett’s metaplasia and wild-type squamous islands, means that these structures warrant further detailed analysis.

In conclusion, we have demonstrated two important features of Barrett’s oesophagus. First, clonal heterogeneity arises from multiple independent clones, previously undetectable by whole biopsy analysis; and second, Barrett’s metaplasia and neo-squamous islands can arise from the oesophageal gland squamous ducts.
